# Spectrum of Congenital Anomalies Among Neonates With Trisomy 21 Born at Salmaniya Medical Complex, Bahrain: A Retrospective Review (2015-2020)

**DOI:** 10.7759/cureus.110758

**Published:** 2026-06-12

**Authors:** Abdulraoof Almadhoob, Maryam Busehail, Hasan Radhi, Maryam Alqayem, Hussain Alshammasi

**Affiliations:** 1 Neonatology, Salmaniya Medical Complex, Manama, BHR; 2 Medical Genetics, Salmaniya Medical Complex, Manama, BHR; 3 Pediatrics, Salmaniya Medical Complex, Manama, BHR; 4 Pediatrics, American Mission Hospital - Bahrain, Manama, BHR

**Keywords:** bahrain, congenital anomalies, down's syndrome, neonatal intensive care unit (nicu), trisomy of 21

## Abstract

Background: Down syndrome (DS) is the most prevalent chromosomal disorder globally and is frequently associated with a complex spectrum of multisystem congenital malformations, including cardiovascular, gastrointestinal, endocrine, and hematological disorders. This study aimed to evaluate the clinical spectrum of congenital malformations among neonates with Trisomy 21 born at Salmaniya Medical Complex (SMC), the primary tertiary referral center in Bahrain.

Methods: A retrospective descriptive study was conducted using total population sampling (census) of 110 infants with a confirmed diagnosis of DS registered in the iSeha electronic system over a five-year period (2015 to January 2020). Clinical data, including demographics, perinatal history, and multisystem comorbidities, were extracted and analyzed using SPSS version 29 (IBM Corp., Armonk, NY, USA).

Results: The cohort consisted of 57.3% male population, with a mean maternal age of 35.47 ± 6.76 years. A significant proportion of the neonates (73.6%) required NICU admission, primarily due to congenital heart disease, poor weight gain, and hypoglycemia. Cardiovascular anomalies represented the most significant clinical burden (78.2%), with patent ductus arteriosus (22.7%) and atrioventricular septal defect (18.2%) being the most frequent. Gastrointestinal and hepatic findings were present in 17.3% of the cohort, most notably imperforate anus (4.5%) and duodenal atresia (3.6%), while neonatal jaundice affected 54.5% of the cohort. Hematological disorders were common, including polycythemia (23.6%) and thrombocytopenia (20.9%). Notably, acute myeloid leukemia (AML) was diagnosed in 4.5% of the cohort.

Conclusion: Neonates with DS in Bahrain exhibit a high prevalence of multisystem congenital anomalies, with cardiovascular and hematological disorders being the most prominent. These findings underscore the critical need for structured, multidisciplinary neonatal screening and specialized care frameworks to optimize clinical outcomes and support parental counselling in the region.

## Introduction

Down syndrome (DS) is the most common chromosomal abnormality occurring in humans and is caused by the presence of all or part of a third copy of chromosome 21 [1]. Trisomy 21 incidence accounts for 1 in 650-1000 live births worldwide [2]. Four chromosomal variations characterize the presentation of Trisomy 21: meiotic nondisjunction (96%), translocation (3-4%), mosaicism (1-2%), and partial trisomy (<1%) [3]. In 1990, the incidence of congenital anomalies was studied in Bahrain accounting for 2.7% of live births, among which Trisomy 21 accounts for 0.9 per 1000 deliveries [4], which is considered relatively small in comparison to the international prevalence of 1.33 per live births [5].

DS may be associated with congenital malformation. This includes cardiovascular, up to 58.6%. Atrioventricular septal defect (AVSD) was the most frequent lesion (40.7%) followed by mixed left to right shunt defects (14.7%) and secundum atrial septal defect (ASD) (11.8%). Ventricular septal defect (VSD) is 10.7%, and 8.5% had patent ductus arteriosus (PDA) [6]. Also, it may be associated with structural gastrointestinal (GI) abnormalities in up to 5%. Common associations like duodenal atresia occurred in 40.6% of cases, followed by imperforated anus in 25% [7]. Thyroid dysfunction is a frequently documented comorbidity in individuals with DS, occurring in approximately 15.6% of cases. Within this group, a distinction is made between compensated hypothyroidism (12.5%) and uncompensated hypothyroidism (3.1%) [8]. In addition to endocrine disorders, this population faces significant hematological challenges. Conditions such as thrombocytopenia and polycythemia are common, and there is a profound predisposition to malignancy; specifically, the risk of developing acute leukemia is increased 12-fold compared to the general population [9].

Despite the well-documented association between Trisomy 21 and a wide array of multisystem congenital anomalies, ranging from complex cardiovascular and GI malformations to endocrine and hematological disorders, there remains a significant lack of contemporary, localized data within the Kingdom of Bahrain. This knowledge gap hinders the ability of clinicians to precisely identify which complications are most prevalent among Bahraini neonates, leaving a critical void in the evidence-based management of this vulnerable population.

This research is of vital importance to both the medical community and the families of children with DS in Bahrain. Furthermore, these findings will empower parents through more accurate prenatal and postnatal counselling, fostering a more informed support system. Ultimately, the study serves as a foundational tool for building a more structured, timely, and patient-specific care framework within Bahrain’s healthcare system, ensuring that clinical interventions are optimized to improve the long-term quality of life for children with Trisomy 21. This study aims to evaluate the spectrum of congenital anomalies among neonates with Trisomy 21 born at Salmaniya Medical Complex (SMC), Bahrain (2015-2020).

## Materials and methods

This retrospective descriptive study was conducted at the SMC located in Manama. It is a premier public healthcare facility and the largest tertiary care hospital in the Kingdom of Bahrain, having evolved into a multidisciplinary medical hub serving as the primary referral center for the country’s population. The study population encompasses all pediatric patients aged 1 day to 14 years with a confirmed diagnosis of DS who were registered in the iSeha system and received follow-up care at SMC. To ensure a comprehensive analysis, the study utilized a total population sampling technique (census), including all confirmed cases identified over a five-year period from January 2015 to January 2020. Based on the data from The Global Economy, the population of the Kingdom of Bahrain reached 1.59 million in 2024, with individuals aged 0-14 accounting for 18.72% of the total population.

This study was approved by the Research & Research Ethics Committee of Government Hospitals on April 20, 2026 (Approval No. GH-04-257). To protect participant privacy, all patient data were fully de-identified and kept strictly confidential.

Data collection involved a detailed review of electronic medical records via the iSeha system and a manual cross-reference of the neonatal registry books for all admissions. The primary investigator (PI) and trained study personnel extracted relevant clinical data, including patient demographics, comorbidities, and comprehensive past medical histories. 

All collected data were managed with strict adherence to patient confidentiality, with information processed and reported in a de-identified, aggregate format. For the analysis phase, data were entered in Microsoft Excel (Microsoft, Redmond, WA, USA) for cleaning and coding, followed by formal statistical analysis using the Statistical Package for the Social Sciences (SPSS) version 29 (IBM Corp., Armonk, NY, USA). This analysis was carried out to identify the prevalence of complications and the clinical progression of DS within the specified pediatric population at SMC. Continuous variables were expressed as mean ± standard deviation (SD), while categorical data were presented as frequencies and percentages.

## Results

Data analysis

Data were entered in Microsoft Excel for cleaning and coding, followed by formal statistical analysis using the Statistical Package for the Social Sciences (SPSS) version 29 (IBM Corp., Armonk, NY, USA). This analysis was carried out to identify the prevalence of complications and the clinical progression of DS within the specified pediatric population at SMC. Continuous variables were expressed as mean ± SD, while categorical data were presented as frequencies and percentages.

Table 1 summarizes the baseline demographic characteristics and growth parameters of the study cohort. This study involved 110 DS infants, with more than half of them being male at 63 (57.3%). The mean age of the patients was 41.70 ± 22.64 (with an average of 0.5 to 89) months, with an average age at the time of initial diagnosis being 14.20 ± 13.66 months, and the mean maternal age was 35.47 ± 6.76 years. At birth, the cohort had an average weight of 2730.2 ± 589.03 grams and a mean head circumference of 31.72 ± 2.02 centimeters.

**Table 1:** Baseline demographics and growth parameters SD: standard deviation.

Variable		Number	Percentage
Gender	Male	63	57.3
	Female	47	42.7
Age	Mean ± SD	41.70 ± 22.64 months	
Age at the time of diagnosis	Mean ± SD	14.20 ±13.66 months	
Maternal age	Mean ± SD	35.47 ± 6.76 years	
Birth weight	Mean ± SD	2730.2 ± 589.03 grams	
Head circumference	Mean ± SD	31.72 ±2.02 centimeters	

Table 2 outlines the perinatal and neonatal history of the participants. Intrauterine growth restriction (IUGR) was observed in 16 (14.5%) of the cohort. The majority of the infants were delivered via normal vaginal delivery, accounting for 64 (58.2%), while 39 (35.5%) required a cesarean section. A highly significant proportion of the neonates, 81 (73.6%), required admission to the neonatal intensive care unit (NICU), though all were successfully discharged alive. Among those admitted to the NICU, the primary specified causes included poor weight gain in 13 (11.8%), congenital heart disease in 12 (10.9%), and hypocalcemia in 10 (9.1%), with a substantial number admitted for other unspecified neonatal complications, accounting for 45 (40.9%).

**Table 2:** Perinatal and neonatal history IUGR: intrauterine growth restriction; NICU: neonatal intensive care unit; C/S: cesarean section; PPHN: persistent pulmonary hypertension of the newborn; CHD: congenital heart disease.

Variable		Frequency	Percentage
IUGR	Yes	16	14.5
	No	94	85.5
Mode of delivery	Normal	64	58.2
	C/S	39	35.5
	Assisted	3	2.7
	Missing data	4	3.6
NICU admission	Yes, and discharged alive	81	73.6
	No	29	26.4
Causes of admission to NICU (n = 81)	Hypoglycemia	10	9.1
	PPHN	1	0.9
	CHD	12	10.9
	Weight gain	13	11.8
	Others	45	40.9

Table 3 details the prevalence and types of cardiovascular system (CVS) abnormalities, highlighting a high incidence of congenital heart defects (CHDs) typical of this population. Overall, 86 (78.2%) patients presented with a documented CVS disease. The most frequently observed cardiac anomaly was PDA, affecting 25 (22.7%) of the cohort, closely followed by AVSD in 20 (18.2%), ASD in 18 (16.4%), and VSD in 15 (13.6%). Tetralogy of Fallot (TOF) was present in 3 (2.7%) cases, while other rare structural or functional anomalies, such as aberrant pulmonary arteries and persistent pulmonary hypertension of the newborn (PPHN), accounted for the remaining 5 (4.5%) of the cardiovascular complications.

**Table 3:** CVS abnormalities CVS: cardiovascular system; ASD: atrial septal defect; VSD: ventricular septal defect; AVSD: atrioventricular septal defect; PDA: patent ductus arteriosus; TOF: tetralogy of Fallot; PFO: patent foramen ovale; PPHN: persistent pulmonary hypertension of the newborn.

Variable		Number	Percentage
Presence of CVS abnormalities	Yes	86	78.2
	No	24	21.8
Major CVS abnormalities (n = 81)	ASD	18	16.4
	VSD	15	13.6
	AVSD	20	18.2
	PDA	25	22.7
	TOF	3	2.7
Other CVS abnormalities (n = 5)	Aberrant pulmonary artery	1	0.9
	Aberrant right pulmonary artery and small PDA	1	0.9
	Mild aortic regurgitation	1	0.9
	PDA, PFO, ASD	1	0.9
	PPHN	1	0.9

Table 4 presents the GI, endocrine, and hepatic findings of the cohort. Endocrine screening revealed abnormal thyroid-stimulating hormone (TSH) levels at birth in 8 (7.3%) of the evaluated neonates. Structural and functional gastrointestinal abnormalities were present in 19 (17.3%) patients, with imperforate anus in 5 (4.5%) and duodenal atresia in 4 (3.6%) being the most prevalent structural defects. Additional GI and hepatic complications included cow's milk protein allergy in 3 (2.7%), mild hepatomegaly with gallbladder pathology in 3 (2.7%), and isolated instances of cholestatic jaundice and meconium plug syndrome. Furthermore, neonatal jaundice was highly prevalent in the immediate postnatal period, affecting over half of the assessed cohort at 60 (54.5%).

**Table 4:** GIT and endocrine abnormalities GIT: gastrointestinal tract; TSH: thyroid-stimulating hormone; CMPA: cow's milk protein allergy; CMV: cytomegalovirus.

Variable		Frequency	Percentage
TSH at birth (n = 106)	Normal	102	92.7
	Abnormal	8	7.3
Presence of GIT abnormalities	Yes	19	17.3
	No	91	82.7
GIT abnormalities (n = 19)	Duodenal atresia	4	3.6
	Umbilical hernia	1	0.9
	Imperforated anus	5	4.5
	Cow's milk protein allergy +(CMPA)	3	2.7
	Mild hepatomegaly and gall bladder conditions	3	2.7
	Cholestatic jaundice	1	0.9
	CMV hepatitis and cholestatic jaundice	1	0.9
	Meconium plug syndrome	1	0.9
Neonatal jaundice (n = 104)	Yes	60	54.5
	No	44	40.0

Table 5 documents the hematological and oncological profiles of the patients. Polycythemia was the most common hematological derangement, identified in 26 (23.6%) of the evaluated patients, followed closely by thrombocytopenia in 23 (20.9%). Oncological and pre-leukemic assessments indicated that 4 (3.6%) of the tested subset experienced transient myeloproliferative disease (TMD), while a leukemoid reaction was noted in a single patient (0.9%). Notably, acute myeloid leukemia (AML) was diagnosed in 5 (4.5%) of the clinically evaluated cohort.

**Table 5:** Hematological and oncological findings AML: acute myeloid leukemia.

Variable		Number	Percentage
Polycythemia (n = 106)	Yes	26	23.6
	No	80	72.7
Leukemoid reaction (n = 102)	Yes	1	0.9
	No	101	91.8
Leukemia (n = 105)	Yes (AML)	5	4.5
	No	100	90.9
Thrombocytopenia (n = 106)	Yes	23	20.9
	No	83	75.5
Transient myeloproliferative disease (n = 71)	Yes	4	3.6
	No	67	60.9

## Discussion

The demographic profile of our cohort was characterized by a mean maternal age of 35.47 ± 6.76 years and a 57.3% male predominance; these results align with established epidemiological patterns, which reported that advanced maternal age remains the most significant non-genetic risk factor for Trisomy 21 [10-12]. Furthermore, these findings are consistent with a 2022 study in China, which demonstrated that while DS occurs across all age groups, incidence rates increase significantly in women ≥34 years [13]. Additionally, the sex distribution aligns with international data; for instance, the United Arab Emirates reported a male-to-female ratio of 1.6 [14], and a study in Brazil showed a male predominance (53.57%) [12]. The study identifies a substantial 73.6% NICU admission rate among infants with DS. These neonates face a heightened risk of requiring intensive care and typically experience significantly longer hospitalizations. This trend is largely driven by a higher incidence of preterm births and complications stemming from syndrome-specific congenital malformations [12]. Several factors unique to Arab populations explain the higher frequency of DS births. First, women in these societies often continue childbearing into their later, high-risk reproductive years, increasing the physiological likelihood of chromosomal anomalies. Second, access to and acceptance of antenatal diagnosis and selective abortion are highly constrained. Across most Arab countries, both national laws and Islamic bioethical frameworks reject disability-based terminations for conditions compatible with life, meaning that even when a prenatal diagnosis is made, the pregnancy is almost universally carried to term.

Cardiovascular malformations represent the most significant clinical burden in our cohort, with a prevalence of 78.2%. These congenital anomalies, alongside prematurity, are the primary drivers of the elevated NICU admission rates and prolonged hospitalizations observed in our study. Our recorded prevalence of 78.2% is significantly higher than that reported in Sudan (43.1%) [15], Libya (45%) [16], Italy (56%) [17], and Oman (60%) [18], yet it remains lower than the rates documented in Nigeria (78%) [19]. Regionally, our results exceed the pooled prevalence from a Saudi Arabian study of 1,590 subjects, which indicated a CHD prevalence of 66.1% (95% CI: 57.2% to 74.5%) among individuals with DS [20]. Globally, our findings are higher than those of a comprehensive pooled analysis of 60,610 individuals, which identified a global CHD prevalence of 49.9% among those with DS [21]. This prevalence appears subject to geographic variation; while Europe reported the lowest rates (46.2%), prevalence in North America (51.6%) and Asia (54.2%) was notably higher [21]. This high prevalence justifies a shift in clinical focus toward early, comprehensive echocardiographic screening for all DS neonates in the region to prevent early-onset pulmonary hypertension.

The distribution of specific cardiac lesions in our cohort presents a unique profile compared to existing regional literature. While PDA emerged as the most frequent defect in our study (22.7%), followed by AVSD at 18.2%, this contrasts with many neighboring countries where AVSD is overwhelmingly predominant. For instance, studies in Oman [18], Nigeria (56.3%) [19], and Sudan (ranging from 32% to 48%) [15,22] all identify AVSD as the primary cardiac abnormality in children with DS. Furthermore, our results differ from findings in Egypt, where VSD is the most common lesion [23], and Libya, where ASD was reported as the most frequent isolated lesion (23%) [16]. This variability across African and Middle Eastern populations suggests that while CHD is a universal hallmark of DS, the specific anatomical manifestation may be influenced by localized genetic or environmental modifiers.

The multisystemic involvement of DS is further evidenced by the high prevalence of GI and endocrine complications observed in this population. In the present study, the prevalence of structural and functional GI defects was 17.3%, with imperforate anus (4.5%) and duodenal atresia (3.6%) identified as the most frequent anomalies. These figures significantly exceed several international benchmarks, such as reports from France, which noted digestive system anomalies in 6% of cases [24], and the United States, where the Atlanta and National Down Syndrome Projects identified a 6.7% prevalence of congenital GI defects [25]. Specifically, the US study found duodenal stenosis/atresia in 3.9% of infants and anal stenosis/atresia in only 1.0% [25], rates notably lower than those found in our cohort. Similarly, research in Brazil indicated that digestive tract malformations occurred in 5% of patients, with duodenal atresia and imperforate anus also being the most common findings among the malformed group [7]. The clinical severity of these conditions is underscored by literature suggesting that infants with DS are 122 times more likely to suffer from the concomitant occurrence of small intestinal atresia and Hirschsprung disease (HD) than the general population [26]. The marked discrepancy between our observed 17.3% prevalence and the 5-7% range reported in Western and South American literature justifies further investigation into potential regional genetic modifiers or environmental factors. Such variations emphasize the importance of tailored clinical surveillance and localized research to better understand the unique phenotypic profile of DS within this specific geographic context.

In the endocrine profile observed in our study, 7.3% of infants presented with abnormal TSH levels at birth. Literature indicates that children with DS face an incidence of congenital hypothyroidism 28 times higher than that of the general population [27]. While global prevalence estimates for congenital hypothyroidism in DS typically range from 1.5% to 6.1% [27], our observed rate of 7.3% underscores a particularly high neonatal endocrine burden in this cohort. Comparative data from pediatric and adult populations further illustrate the progressive nature of thyroid dysfunction in DS. In Turkey, a study of 320 children reported that 28.1% exhibited abnormal thyroid function, with a notable 25.3% of those cases identified as compensated hypothyroidism [28]. Similarly, a multi-institutional registry study in the United States identified a 19.0% prevalence of newly diagnosed thyroid disorders during specialty clinic visits [27]. By adulthood, the prevalence appears to climb even higher; research in England involving 106 adults revealed a cumulative endocrine dysfunction rate of 40.6%, with nearly 35% of cases being newly identified through screening [29]. These findings underscore the early-onset and persistent nature of endocrine dysfunction in individuals with DS. The high prevalence of subclinical and late-onset issues identified during the first decade of life and the subsequent increase into adulthood justify the implementation of mandatory, repeat thyroid screening throughout the first year of life and beyond. 

The hematological and oncological profile of our cohort highlights the unique clinical risks associated with Trisomy 21, specifically through the high observed rates of polycythemia (23.6%) and thrombocytopenia (20.9%). These findings contrast with the study by Vlad et al., who reported a 58% overall anomaly rate characterized predominantly by leukopenia (17.6%), anemia (16.9%), and macrocytosis (16.9%), with a significantly lower frequency of thrombocytopenia (3.67%) [30]. Most importantly, no cases of acute lymphocytic leukemia (ALL) were recorded in our study, while a 4.5% prevalence of acute myeloid leukemia (AML) was reported, which exceeds the 1.47% incidence reported by Vlad et al. and the hematological manifestation rate seen in an Indian study of 239 cases, which identified four cases of transient myeloproliferative disorder (TMD) and four of acute leukemia [30,31]. These elevated rates of neonatal abnormalities align with established literature identifying transient neutrophilia, thrombocytopenia, and polycythemia as common features of constitutional Trisomy 21 [32]. Furthermore, while TMD affects up to 30% of neonates and often regresses spontaneously, the 20% risk of progression to myeloid leukemia underscores the necessity for the vigilant hematological monitoring observed in our clinical practice [32,33].

Strength and limitations of the study

A key strength of this study lies in its comprehensive total population sampling (census) approach, which utilized a five-year longitudinal dataset from Bahrain's primary tertiary referral center, SMC. This extensive timeframe, combined with a rigorous dual-source verification method involving the iSeha electronic system and manual neonatal registries, ensured high data accuracy and a representative clinical profile of Trisomy 21 in the Kingdom. However, the study is subject to several limitations, primarily its retrospective design, which introduces the potential for inconsistent documentation and missing clinical data. While the study was conducted at the nation’s leading referral hub, its single-center scope may not capture infants managed exclusively in the private sector, potentially impacting the reported national prevalence. Additionally, the cohort size of 110 infants limits the ability to perform deep statistical sub-analyses on rarer anomalies, and the lack of specific genetic subtyping prevents a more detailed correlation between chromosomal mechanisms and clinical phenotypes. Finally, the scope was restricted to the neonatal period, meaning long-term developmental outcomes were not evaluated.

## Conclusions

The study concludes that neonates with DS in Bahrain experience a significant clinical burden, with a large majority requiring intensive care for multi-system anomalies. Cardiovascular malformations, such as PDA and AVSDs, are the most frequent complications, alongside common hematological disorders like polycythemia and thrombocytopenia. These results underscore the need for a standardized, multidisciplinary screening protocol at birth that includes universal neonatal echocardiography and regular blood monitoring. Additionally, establishing a national DS registry is recommended to ensure effective longitudinal follow-up and provide families with an evidence-based roadmap for their children's long-term care.
